# Supplementary material for: The effects of N-acetyl cysteine on intrinsic functional connectivity and neural alcohol cue reactivity in treatment-seeking individuals with alcohol use disorder: a preliminary study
Source: Psychopharmacology (Berl). 2024 Aug 5;242(1):149–60. doi: 10.1007/s00213-024-06656-z (PMC11742866; doi:10.1007/s00213-024-06656-z)
Supplement: Supplementary file 1 — Supplementary Material 1 [file 213_2024_6656_MOESM1_ESM.pdf]

The effects of N-acetyl Cysteine on intrinsic functional connectivity and neural alcohol cue reactivity in treatment-seeking individuals with alcohol use disorder: a preliminary study

Warren B Logge, PhD [1,2*], Paul S Haber PhD [1,2,3], Tristan P Hurzeler, MBMS [1,2], Ellen E Towers, MBMS [1,2], Kirsten C Morley, PhD [1,2]

[1] Edith Collins Centre for Translational Research in Alcohol, Drugs and Toxicology, Royal Prince Alfred Hospital, Sydney Local Health District, NSW, Australia

[2] Specialty of Addiction Medicine, Central Clinical School, Faculty of Medicine and Health, University of Sydney, NSW, Australia.

[3] Drug Health Services, Sydney Local Health District, NSW, Australia

Supplementary Material

Methods

Participants

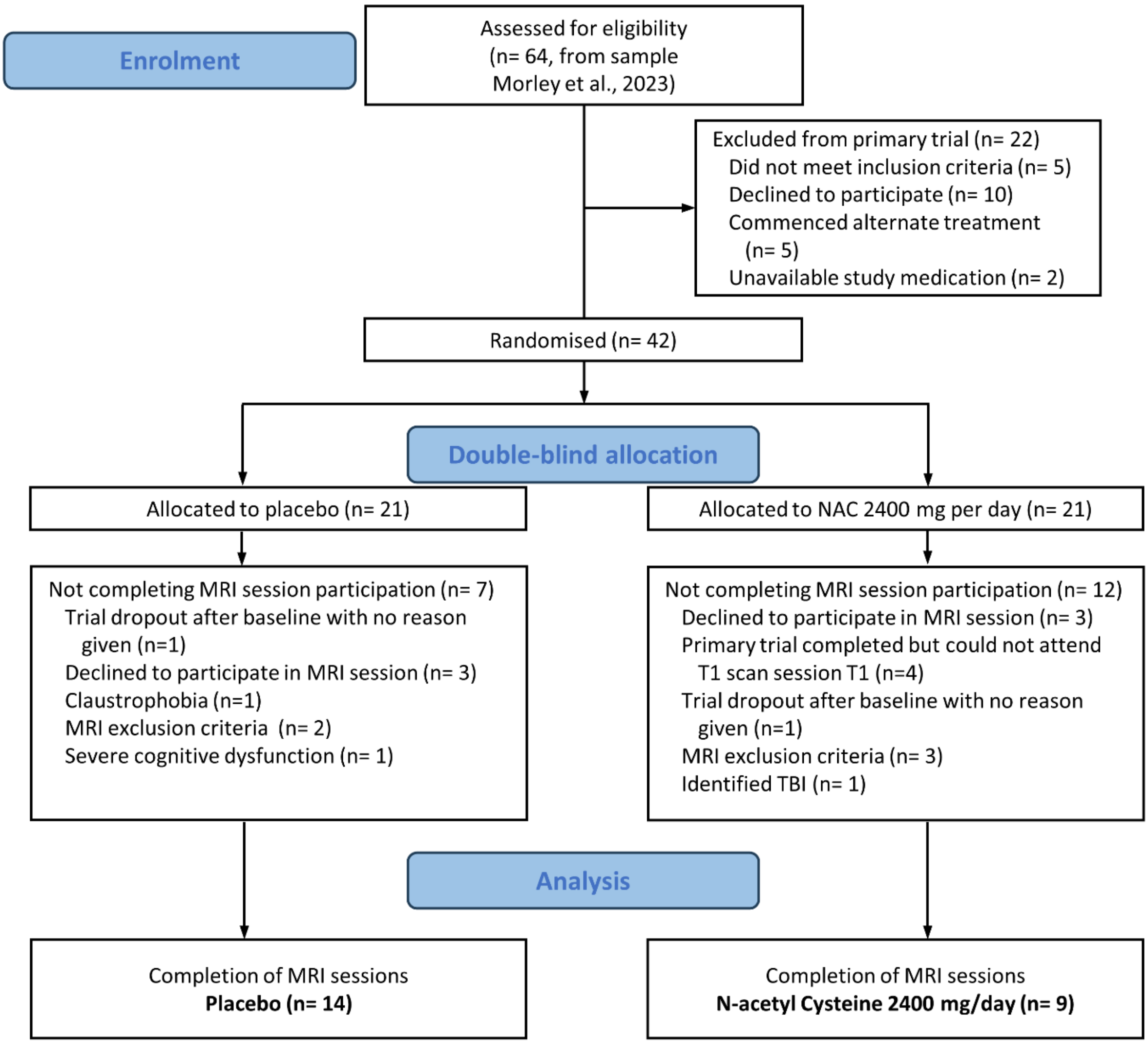

Supplementary Figure 1 CONSORT flow diagram of participant recruitment and randomization

Image processing

Anatomical data preprocessing

A total of 2 T1-weighted (T1w) images were found within the input BIDS dataset. All of them were corrected for intensity non-uniformity (INU) with N4BiasFieldCorrection (Tustison et al., 2010), distributed with ANTs 2.3.3 (Avants et al., 2008), RRID:SCR_004757). The T1w-reference was then skull-stripped with a Nipype implementation of the antsBrainExtraction.sh workflow (from ANTs), using OASIS30ANTs as target template. Brain tissue segmentation of cerebrospinal fluid (CSF), white-matter (WM) and gray-matter (GM) was performed on the brain-extracted T1w using fast (FSL 5.0.9, RRID:SCR_002823, (Zhang et al., 2001). A T1w-reference map was computed after registration of 2 T1w images (after INU-correction) using mri_robust_template (FreeSurfer 6.0.1, (Reuter et al., 2010). Brain surfaces were reconstructed using recon-all (FreeSurfer 6.0.1, RRID:SCR_001847, (Dale et al., 1999), and the brain mask estimated previously was refined with a custom variation of the method to reconcile ANTs-derived and FreeSurfer-derived segmentations of the cortical gray-matter of Mindboggle (RRID:SCR_002438, (Klein et al., 2017). Volume-based spatial normalization to two standard spaces (MNI152NLin2009cAsym, MNI152NLin6Asym) was performed through nonlinear registration with antsRegistration (ANTs 2.3.3), using brain-extracted versions of both T1w reference and the T1w template. The following templates were selected for spatial normalization: ICBM 152 Nonlinear Asymmetrical template version 2009c (Fonov et al., 2009), RRID:SCR_008796; TemplateFlow ID: MNI152NLin2009cAsym], FSL’s MNI ICBM 152 non-linear 6th Generation Asymmetric Average Brain Stereotaxic Registration Model((Evans et al., 2012), RRID:SCR_002823; TemplateFlow ID: MNI152NLin6Asym).

Functional data preprocessing

For each of the 2 BOLD runs found per subject (across all tasks and sessions), the following preprocessing was performed. First, a reference volume and its skull-stripped version were generated using a custom methodology of fMRIPrep. A B0-nonuniformity map (or fieldmap) was estimated based on two (or more) echo-planar imaging (EPI) references with opposing phase-encoding directions, with 3dQwarp (Cox and Hyde, 1997) (AFNI 20160207). Based on the estimated susceptibility distortion, a corrected EPI (echo-planar imaging) reference was calculated for a more accurate co-registration with the anatomical reference. The BOLD reference was then co-registered to the T1w reference using bbregister (FreeSurfer) which implements boundary-based registration(Greve and Fischl, 2009). Co-registration was configured with six degrees of freedom. Head-motion parameters with respect to the BOLD reference (transformation matrices, and six corresponding rotation and translation parameters) are estimated before any spatiotemporal filtering using mcflirt (FSL 5.0.9,(Jenkinson et al., 2002)). BOLD runs were slice-time corrected using 3dTshift from AFNI 20160207 ((Cox and Hyde, 1997)x, RRID:SCR_005927). The BOLD time-series (including slice-timing correction when applied) were resampled onto their original, native space by applying a single, composite transform to correct for head-motion and susceptibility distortions. These resampled BOLD time-series will be referred to as preprocessed BOLD in original space, or just preprocessed BOLD. The BOLD time-series were resampled into standard space, generating a preprocessed BOLD run in MNI152NLin2009cAsym space. First, a reference volume and its skull-stripped version were generated using a custom methodology of fMRIPrep. All resamplings can be performed with a single interpolation step by composing all the pertinent transformations (i.e. head-motion transform matrices, susceptibility distortion correction when available, and co-registrations to anatomical and output spaces). Gridded (volumetric) resamplings were performed using

antsApplyTransforms (ANTs), configured with Lanczos interpolation to minimize the smoothing effects of other kernels (Lanczos 1964 (Lanczos, 1964)). Non-gridded (surface) resamplings were performed using mri_vol2surf (FreeSurfer).

Resting state post-processing of fmriprep outputs

The eXtensible Connectivity Pipeline (XCP) (Ciric et al., 2017; Satterthwaite et al., 2013) was used to post-process the outputs of fMRIPrep version 20.2.7 (Esteban et al., 2020; Esteban et al., 2019), RRID:SCR_016216). XCP was built with Nipype 1.8.5 (Gorgolewski et al. 2011, RRID:SCR_002502). For each of the two BOLD runs found per subject (across all tasks and sessions), the following post-processing was performed. In order to identify high-motion outlier volumes, framewise displacement was calculated using the formula from Power et al. (2014), with a head radius 40.0 mm. Volumes with framewise displacement greater than 0.4 mm were flagged as high-motion outliers for the sake of later censoring (Power et al., 2014). In total, 36 nuisance regressors were selected from the preprocessing confounds, according to the '36P' strategy. These nuisance regressors included six motion parameters, mean global signal, mean white matter signal, mean CSF signal with their temporal derivatives, and the quadratic expansion of six motion parameters, tissues signals and their temporal derivatives (Ciric et al., 2017; Satterthwaite et al., 2013). Finally, linear trend and intercept terms were added to the regressors prior to denoising. The BOLD data were despiked with 3dDespike. Nuisance regressors were regressed from the BOLD data using linear regression, as implemented in nilearn 0.10.0 (Abraham et al., 2014). Any volumes censored earlier in the workflow were then interpolated in the residual time series produced by the regression. The interpolated timeseries were then band-pass filtered using a(n) second-order Butterworth filter, in order to retain signals within the 0.01-0.08 Hz frequency band. The filtered, interpolated time series were then re-censored to remove high-

motion outlier volumes. The denoised BOLD was smoothed using Nilearn with a Gaussian kernel (FWHM=6.0 mm).

Processed functional timeseries were extracted from the residual BOLD signal with Nilearn's (version 0.10.0, (Abraham et al., 2014)) NiftiLabelsMasker was used for the Schaefer 17-network 400 parcel atlas (Schaefer et al., 2018). Corresponding pair-wise functional connectivity between all regions was computed for each atlas, which was operationalized as the Pearson's correlation of each parcel's unsmoothed timeseries. In cases of partial coverage, uncovered voxels (values of all zeros or NaNs) were either ignored, when the parcel had >50.0% coverage, or were set to zero, when the parcel had <50.0% coverage.

Many internal operations of XCP use AFNI (Cox, 1996; Cox and Hyde, 1997), ANTS (Avants et al., 2009), TemplateFlow version 0.8.1 (Ciric et al., 2022), matplotlib version 3.4.3 (Hunter, 2007), Nibabel version 5.0.1 (Brett et al. 2022), Nilearn version 0.10.0 (Abraham et al., 2014), numpy version 1.22.4 (Harris et al., 2020), pybids version 0.15.5 (Yarkoni et al. 2019) (Yarkoni et al., 2019), and scipy version 1.9.1(Virtanen et al., 2020). For more details, see the xcp_d website https://xcp-d.readthedocs.io.

Regions of Interest

Regions of interest (ROIs) for assessing cue reactivity brain acitivation were selected based on areas correlated with alcohol cue reactivity identified by meta-analyses evaluating cue reactivity and pharmacotherapy studies in AUD (Courtney et al, 2016, Schacht et al., 2013, Zeng et al., 2021) comprise key regions in drug cue reactivity associated with motivational drives and regulation of motivation and salience of drug cues considered to be most likely to show responsivity to alcohol cues, while reducing multiple comparisons. Five ROIs were used: the left and right caudate, the left and right dorsolateral prefrontal cortex (DLPFC), and bilateral ventromedial prefrontal cortex (VMPFC). The caudate was defined

here as caudate body from the Harvard-Oxford subcortical probability atlas (http://www.cma.mgh.harvard.edu/fsl_atlas.html). As previous studies implementing in functional regions of interest within AUD treatment utilized probabilistic ROIs , including the only other study evaluating cue reactivity in substance use (Schulte et al., 2019), we implemented probabilistic maps for the DLPFC and VMPFC defined using the Brainmap database (Fox and Lancaster, 2002). This involved utilizing BrainMap Sleuth and GingerAle software, as previously utilized in studies evaluating treatment effects in substance use, including NAC in cocaine use disorder (Schulte et al., 2019) and baclofen in AUD (Holla et al., 2018).

The search function in BrainMap Sleuth was used to identify relevant studies. Papers containing significant activation results for the DLPFC and VMPFC were selected for relevant contrasts. Talairach coordinates from the selected studies were exported to GingerAle. Probabilistic voxel maps were created using a P-value threshold. For the VMPFC, clusters were created with a $P \geq .90$ threshold which provided a smaller and more precise ROI. Initial clusters were in Talairach space and were later repeated in MNI space for standardization using MarsBar toolbox. Initial clusters for the DLPFC were created in MNI space. The large initial clusters were refined by applying an ROI filter to exclude medial/superior areas with a threshold of $\geq .80$. A second pass utilized a higher $P \geq .90$ threshold to create smaller, more precise ROIs. The final DLPFC ROIs were labeled and stored in the MarsBar toolbox. To improve specificity, both ROIs were masked with gray matter probability maps supplied within the SPM12 toolbox (TPM 1). Comparisons between gray matter-only ROIs and non-transformed ROIs showed that gray matter masking resulted in more activation being reported.

The ROIs are visualized in Supplementary Figure 2 showing the location of the five ROIs, and the volume of the ROIS were as follows: left caudate = 5536 mm^3, right caudate = 5384 mm^3, left DLPFC = 21560 mm^3, right DLPFC = 8612 mm^3, bilateral VMPFC = 40512 mm^3.

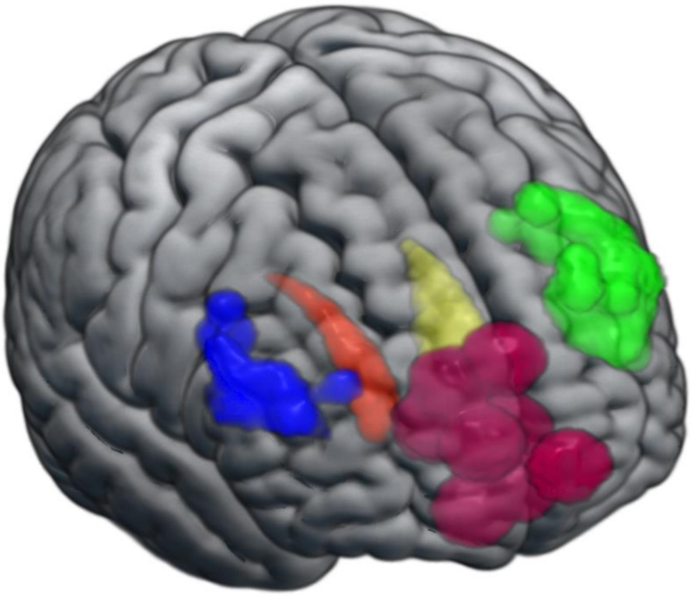

Supplementary Figure 2. Visualisation of extracted brain Regions of interest (ROIs), displayed on 3D MNI template. Brain shown with anterior-facing view with right side shown nearest. Brain regions are colour-coded. Left (yellow) and right (orange) caudate body ROIs constructed from the Harvard-Oxford Subcortical Atlas; probabilistic ROIs constructed for functional ROIs left (green) and right (blue) dorsolateral prefrontal cortex, and ventromedial prefrontal cortex (magenta) using the Brainmap database (Fox & Lancaster, 2002), threshold = ≥.90.

Supplementary Results

Supplementary Table 1 Linear Mixed Effects models for AUQ

	T0 (Baseline)			T1 (During Treatment)			T0 vs T1 Full model		
Predictors	β	CI	p	β	CI	p	β	CI	p
(Intercept)	31.89	13.89 – 49.88	**.001**	33.84	18.80 – 48.88	**<.001**	29.86	14.90 – 44.82	**<.001**
Treatment (NAC)	-3.52	-12.18 – 5.13	.425	-5.97	-13.21 – 1.26	.105	-2.23	-10.49 – 6.02	.596
Pre/Post Scan (Post)	5.27	-2.14 – 12.68	.164	-5.52	-17.10 – 6.07	.35	1.26	-12.70 – 15.22	.86
Age	-0.28	-0.64 – 0.07	.12	-0.33	-0.63 – -0.03	**.029**	-0.24	-0.53 – 0.05	.104
Treatment (NAC) * Pre/Post Scan (Post)	0.19	-3.37 – 3.75	.916	-0.63	-6.20 – 4.94	.825	-0.64	-9.56 – 8.28	.888
Pre/Post Scan * age	-0.09	-0.24 – 0.05	.216	0.12	-0.11 – 0.35	.311	-0.01	-0.28 – 0.26	.942
Session (T1)							-0.43	-5.98 – 5.12	.88
Session (T1) * Treatment (NAC)							-0.68	-9.55 – 8.19	.88
Session * Pre/Post Scan							-0.57	-8.42 – 7.28	.887
Session * Treatment * Pre/Post Scan							-0.65	-13.20 – 11.90	.919
**Random Effects**									
σ^2	8.32			20.35			56.13		
τ_{00}	89.87 ID			48.29 ID			39.49 ID		
ICC	0.92			0.7			0.41		
N	22 ID			22 ID			23 ID		
Observations	44			44			92		
Marginal R^2 / Conditional R^2	0.167 / 0.929			0.245 / 0.776			0.115 / 0.480		

Note. Reference category for predictor shown in brackets. σ^2 = random effects variance; τ_{00} = random intercept variance ICC = intra-class correlation coefficient; ID = individual participants (random factor).

Alcohol Cue Reactivity

Supplementary Table 2 Linear Mixed Effects Sensitivity Analysis Model for ALC contrast across 5 ROIs

	Bl VMPFC			L Caudate			R Caudate			L DLPFC			R DLPFC		
Predictors	β	CI	p	β	CI	p	β	CI	p	β	CI	p	β	CI	p
(Intercept)	0.46	-1.41–2.33	0.618	-0.25	-1.91–1.40	0.757	-0.13	-1.57–1.31	0.858	-0.43	-1.33–0.46	0.332	-0.41	-1.50–0.68	.449
Session	-0.37	-0.86–0.12	0.137	-0.46	-1.34–0.41	0.289	-0.55	-1.31–0.21	0.149	-0.29	-0.68–0.09	0.131	-0.54	-1.11–0.03	.061
Treatment	-0.22	-1.19–0.74	0.64	-0.76	-1.79–0.27	0.141	-0.75	-1.64–0.14	0.097	-0.03	-0.55–0.48	0.904	0.1	-0.58–0.77	.771
Age	0	-0.04–0.03	0.898	0.01	-0.02–0.04	0.515	0.01	-0.02–0.03	0.587	0.01	-0.01–0.03	0.218	0.01	-0.01–0.03	.27
Antidepressant Use	-0.11	-1.00–0.79	0.809	0.54	-0.23–1.30	0.164	0.49	-0.17–1.15	0.143	-0.05	-0.47–0.37	0.813	-0.21	-0.71–0.29	.405
Alcoholic Liver Disease	-0.36	-1.45–0.72	0.503	0.13	-0.80–1.07	0.771	-0.09	-0.90–0.72	0.821	-0.07	-0.58–0.44	0.795	0.18	-0.44–0.79	.563
Smoker	0.5	-0.68–1.68	0.395	0.03	-0.99–1.05	0.95	0.15	-0.73–1.04	0.726	0.52	-0.03–1.08	0.064	0.38	-0.29–1.05	.252
Last Drinking Day	-0.01	-0.05–0.03	0.669	0.02	-0.03–0.08	0.371	0.02	-0.03–0.07	0.481	0	-0.03–0.02	0.809	0	-0.04–0.04	.928
Session * Treatment	0.03	-0.74–0.81	0.929	0.12	-1.27–1.51	0.862	0.39	-0.82–1.60	0.518	0.15	-0.47–0.76	0.634	0.04	-0.86–0.95	.921
**Random Effects**															
σ^2	0.4			1.28			0.96			0.25			0.55		
τ_{00}	0.67 ID			0.00 ID			0.00 ID			0.07 ID			0.00 ID		
ICC	0.63									0.21			0.01		
N	23 ID			23 ID			23 ID			23 ID			23 ID		
Observations	46			46			46			46			46		
Marginal R^2	0.063			0.169			0.152			0.169			0.19		

P-threshold < .029 (Bonferroni's adjustment corrected)

Note. Reference category for predictor shown in brackets. σ^2 = random effects variance; τ_{00} = random intercept variance ICC = intra-class correlation coefficient; ID = individual participants (random factor); Bl = bilateral, L = left, R = right, DLPFC = dorsolateral prefrontal cortex.

Supplementary Table 3 4 Linear Mixed Effects Sensitivity Analysis Model for ALC contrast across 5 ROIs

	Bl VMPFC			L Caudate			R Caudate			L DLPFC			R DLPFC		
Predictors	β	CI	p	β	CI	p	β	CI	p	β	CI	p	β	CI	p
(Intercept)	0.19	-1.60–1.98	.833	-0.12	-1.55–1.31	.863	-0.04	-1.29–1.20	.943	-0.41	-1.20–0.39	.309	-0.33	-1.28–0.61	.480
Session	-0.21	-0.71–0.29	.400	-0.47	-1.22–0.29	.217	-0.55	-1.20–0.11	.100	-0.21	-0.56–0.13	.216	-0.41	-0.91–0.09	.105
Treatment	-0.14	-1.07–0.79	.759	-0.86	-1.75–0.02	.056	-0.83	-1.60–-0.06	.036	-0.03	-0.49–0.43	.885	0.11	-0.48–0.69	.719
Age	0	-0.04–0.03	.900	0.01	-0.02–0.03	.509	0.01	-0.02–0.03	.579	0.01	-0.00–0.02	.177	0.01	-0.01–0.03	.265
Antidepressant Use	0.04	-0.82–0.89	.931	0.63	-0.04–1.29	.063	0.55	-0.02–1.13	.059	0.01	-0.36–0.38	.950	-0.16	-0.59–0.28	.473
Alcoholic Liver Disease	-0.48	-1.52–0.56	.357	-0.01	-0.81–0.80	.987	-0.17	-0.87–0.53	.620	-0.17	-0.63–0.28	.451	0	-0.53–0.54	.985
Smoker	0.44	-0.69–1.57	.433	0.04	-0.84–0.92	.920	0.17	-0.60–0.93	.659	0.42	-0.07–0.92	.092	0.35	-0.23–0.94	.225
Last Drinking Day	0	-0.04–0.05	.826	0.02	-0.03–0.07	.350	0.01	-0.03–0.05	.565	0	-0.02–0.03	.837	0.01	-0.03–0.04	.702
Session * Treatment	-0.13	-0.92–0.66	.748	0.07	-1.13–1.27	.903	0.4	-0.65–1.44	.446	0.03	-0.52–0.58	.908	-0.01	-0.81–0.78	.971
**Random Effects**															
σ^2	0.41			0.95			0.72			0.2			0.42		
τ_{00}	0.59 ID			0.00 ID			0.00 ID			0.25 ID			0.00 ID		
ICC	0.59									0.21			0.01		
N	23 ID			23 ID			23 ID			23 ID			23 ID		
Observations	46			46			46			46			46		
Marginal R^2	0.058			0.232			0.201			0.148			0.171		

P-threshold < .029 (Bonferroni's adjustment corrected)

Note. Reference category for predictor shown in brackets. σ^2 = random effects variance; τ_{00} = random intercept variance ICC = intra-class correlation coefficient; ID = individual participants (random factor); Bl = bilateral, L = left, R = right, DLPFC = dorsolateral prefrontal cortex.

Supplementary Table 5 Effect sizes for Linear Mixed Effects model for ALC contrast across 5 ROIs

	Bl VMPFC		L Caudate		R Caudate		L DLPFC		R DLPFC	
	Inc. R^2	CI	Inc. R^2	CI	Inc. R^2	CI	Inc. R^2	CI	Inc. R^2	CI
Session	.033	0 – 0.142	.045	0.001 – 0.213	.052	0 – 0.209	.043	0 – 0.201	.122	0.01 – 0.324
Treatment	.008	0 – 0.192	.069	0.001 – 0.254	.047	0 – 0.217	.006	0 – 0.146	.003	0 – 0.112
Age	.002	0 – 0.176	.000	0 – 0.089	.000	0 – 0.093	.034	0 – 0.209	.025	0 – 0.166
Antidepressant Use	.006	0 – 0.194	.022	0 – 0.16	.026	0 – 0.184	.000	0 – 0.102	.004	0 – 0.109
Alcoholic Liver Disease	.004	0 – 0.16	.032	0 – 0.18	.012	0 – 0.132	.014	0 – 0.165	.026	0 – 0.171
Session * Treatment	.018	0 – 0.161	.066	0.001 – 0.244	.036	0 – 0.191	.000	0 – 0.095	.017	0 – 0.134

Note. Inc. R^2 = Inclusive R^2.

Supplementary Table 6 Effect sizes for Linear Mixed Effects model for CON contrast across 5 ROIs

	Bl VMPFC		L Caudate		R Caudate		L DLPFC		R DLPFC	
	Inc. R^2	CI	Inc. R^2	CI	Inc. R^2	CI	Inc. R^2	CI	Inc. R^2	CI
Session	0.022	0 –0.117	0.059	0.001 –0.231	0.059	0.001 –0.232	0.046	0 –0.198	0.112	0.006 –0.314
Treatment	0.006	0 –0.191	0.107	0.007 –0.285	0.069	0.002 –0.25	0.002	0 –0.138	0.003	0 –0.11
Age	0.002	0 –0.161	0.000	0 –0.086	0.000	0 –0.097	0.043	0 –0.243	0.029	0 –0.192
Antidepressant Use	0.001	0 –0.191	0.032	0 –0.175	0.040	0 –0.198	0.000	0 –0.121	0.005	0 –0.112
Alcoholic Liver Disease	0.010	0 –0.185	0.024	0 –0.164	0.008	0 –0.11	0.001	0 –0.119	0.007	0 –0.119
Session * Treatment	0.021	0 –0.157	0.100	0.006 –0.281	0.047	0.001 –0.212	0.005	0 –0.12	0.018	0 –0.153

Note. Inc. R^2 = Inclusive R^2.

Exploratory Whole Brain Analyses

Across the whole sample, irrespective of treatment group, the Alcohol images elicited increased BOLD activation compared to the Control images during fMRI cue reactivity. This was seen in 5 clusters, including one encompassing the left parahippocampal gyrus and fusiform gyrus ($P_{FWE\text{-}corr} < .030$, 280 voxels) and one cluster including the right parahippcampal and fusiform gyrus ($P_{FWE\text{-}corr} < .014$, 454 voxels). Two clusters were observed within the occipital cortex and adjacent regions, primarily the left supramarginal gyrus, angular gyrus, and middle temporal gyrus($P_{FWE\text{-}corr} < .023$, 318 voxels), the right angular gyrus and supramarginal gyrus ($P_{FWE\text{-}corr} < .038$, 237 voxels). One cluster encompassed the posterior cingulate and left precuneus ($P_{FWE\text{-}corr} < .030$, 277 voxels).

No other main effects of time or treatment group were found, and no main effects of covariates. No two-way interactions were seen between condition, time, treatment group were seen. There was a significant three-way interaction of condition, time, and antidepressant use in a cluster that spanned the bilateral thalamus, and right medial dorsal nucleus of the thalamus, extra-nuclear, and extending into the left middle and superior temporal gyri and left insula ($P_{FWE\text{-}corr} < .013$, 682 voxels).

ALC > CON contrasts at T0

Results are presented in Supplementary Table 7. There were no significant effects observed at T0 for any of the 5 ROIs according to any of the variables including treatment group, drinks per drinking day, age, ArLD, or antidepressant use, or any two-way interactions (*p*'s > .0413), indicating that there were no differences in cue reactivity at baseline according to treatment group.

ALC > CON contrasts at T1

Results are presented in Supplementary Table 7. At T1 no significant main treatment effect seen for the ROIs. There was a significant effect for covariates, with a significant main effect of presence of alcoholic liver disease for the right DLPFC, with those with ArLD showing increased alcohol cue reactivity overall (p =.024). There was also a main effect of antidepressant use with those on antidepressants showing increased alcohol cue reactivity (p = .013).

Supplementary Table 7 ANCOVAs for ALC > CON contrast across 5 ROIs during T0 and T1

Predictors	Bl VMPFC	L Caudate Body	R Caudate Body	L DLPFC	R DLPFC
T0					
Treatment	F(1,15)=1.2, p = .292	F(1,15)=0.15, p = .704	F(1,15)=0.13, p = .724	F(1,15)=0.02, p = .878	F(1,15)=0.04, p = .841
Age	F(1,15)=2.59, p = .129	F(1,15)=0.22, p = .646	F(1,15)=0.25, p = .627	F(1,15)=0.09, p = .769	F(1,15)=1.38, p = .258
Alcoholic Liver Disease (Yes)	F(1,15)=3.66, p = .075	F(1,15)=0.1, p = .762	F(1,15)=0.12, p = .737	F(1,15)=0.44, p = .518	F(1,15)=0.4, p = .536
Antidepressant Use (Yes)	F(1,15)=0, p = .99	F(1,15)=0.53, p = .477	F(1,15)=0.91, p = .356	F(1,15)=0.14, p = .718	F(1,15)=1.56, p = .231
Drinks per drinking day	F(1,15)=1.08, p = .315	F(1,15)=0.01, p = .922	F(1,15)=0.21, p = .657	F(1,15)=0.18, p = .674	F(1,15)=0.01, p = .913
Treatment * Age	F(1,15)=0.02, p = .884	F(1,15)=0.19, p = .668	F(1,15)=0.3, p = .595	F(1,15)=0, p = .991	F(1,15)=0.04, p = .844
T1					
Treatment	F(1,14)=0.27, p = .614	F(1,14)=1.6, p = .227	F(1,14)=0.08, p = .776	F(1,14)=2.01, p = .178	F(1,14)=0.18, p = .682
Age	F(1,14)=0, p = .954	F(1,14)=0, p = .971	F(1,14)=0, p = .99	F(1,14)=4.54, p = .051	F(1,14)=6.04, p = **.028**
Alcoholic Liver Disease (Yes)	F(1,14)=2.25, p = .156	F(1,14)=0.05, p = .834	F(1,14)=0, p = .983	F(1,14)=2.96, p = .107	F(1,14)=1.1, p = .312
Antidepressant Use (Yes)	F(1,14)=0.03, p = .865	F(1,14)=0.79, p = .389	F(1,14)=0.19, p = .667	F(1,14)=0, p = .979	F(1,14)=0.39, p = .541
Drinks per drinking day	F(1,14)=2.46, p = .139	F(1,14)=8.73, p = **.011**	F(1,14)=1.74, p = .208	F(1,14)=4.59, p = .05	F(1,14)=0, p = .959
Treatment Days	F(1,14)=1.78, p = .203	F(1,14)=4.78, p = .046	F(1,14)=1.36, p = .263	F(1,14)=3.8, p = .072	F(1,14)=1.98, p = .182
Treatment * Age	F(1,14)=0.87, p = .366	F(1,14)=0.42, p = .527	F(1,14)=0.36, p = .559	F(1,14)=2.89, p = .111	F(1,14)=0.57, p = .462

Supplementary Table 8 Sensitivity Analysis ANCOVAs for ALC > CON contrast across 5 ROIs during T0 and T1

Predictors	Bl VMPFC	L Caudate Body	R Caudate Body	L DLPFC	R DLPFC
T0					
Treatment	F(1,13)=0.61, p=0.447	F(1,13)=0.3, p=0.591	F(1,13)=0.33, p=0.575	F(1,13)=0.18, p=0.678	F(1,13)=0.02, p=0.895
Age	F(1,13)=2.17, p=0.163	F(1,13)=0.03, p=0.875	F(1,13)=0.05, p=0.833	F(1,13)=1.83, p=0.198	F(1,13)=0.02, p=0.9
Alcoholic Liver Disease (Yes)	F(1,13)=3.58, p=0.079	F(1,13)=4.41, p=0.054	F(1,13)=4.83, p=0.045	F(1,13)=5.14, p=0.04	F(1,13)=4.31, p=0.057
Antidepressant Use (Yes)	F(1,13)=0.53, p=0.479	F(1,13)=0.43, p=0.522	F(1,13)=0.44, p=0.518	F(1,13)=1.81, p=0.2	F(1,13)=0.59, p=0.456
Drinks per drinking day	F(1,13)=3, p=0.105	F(1,13)=0.01, p=0.924	F(1,13)=0.26, p=0.621	F(1,13)=3.98, p=0.066	F(1,13)=5.48, p=0.035
Smoker	F(1,13)=0.03, p=0.864	F(1,13)=0.21, p=0.652	F(1,13)=0.16, p=0.699	F(1,13)=1.97, p=0.182	F(1,13)=0.27, p=0.608
Last Drinking Day	F(1,13)=0.04, p=0.841	F(1,13)=0.9, p=0.36	F(1,13)=1.82, p=0.199	F(1,13)=0.91, p=0.356	F(1,13)=0.51, p=0.488
Treatment * Age	F(1,13)=1, p=0.335	F(1,13)=3.3, p=0.091	F(1,13)=0.52, p=0.481	F(1,13)=0, p=0.993	F(1,13)=0.12, p=0.734
T1					
Treatment	F(1,14)=0.42, p=0.526	F(1,14)=0.13, p=0.726	F(1,14)=1.2, p=0.293	F(1,14)=0.19, p=0.672	F(1,14)=0.32, p=0.579
Age	F(1,14)=3.96, p=0.068	F(1,14)=1.17, p=0.298	F(1,14)=1.21, p=0.291	F(1,14)=0.07, p=0.789	F(1,14)=2.12, p=0.169
Alcoholic Liver Disease (Yes)	F(1,14)=0.47, p=0.505	F(1,14)=0.07, p=0.795	F(1,14)=0.01, p=0.93	F(1,14)=4.56, p=0.052	F(1,14)=5.61, p=0.034
Antidepressant Use (Yes)	F(1,14)=2.3, p=0.154	F(1,14)=1.65, p=0.221	F(1,14)=0.74, p=0.404	F(1,14)=3.3, p=0.092	F(1,14)=7.11, p=0.019
Drinks per drinking day	F(1,14)=0.01, p=0.925	F(1,14)=0.48, p=0.502	F(1,14)=0.26, p=0.621	F(1,14)=0.18, p=0.676	F(1,14)=0.16, p=0.698
Treatment Days	F(1,14)=0.63, p=0.442	F(1,14)=0.14, p=0.714	F(1,14)=0.27, p=0.61	F(1,14)=1.61, p=0.227	F(1,14)=3.25, p=0.094
Smoker	F(1,14)=0.74, p=0.406	F(1,14)=0.19, p=0.669	F(1,14)=0.04, p=0.839	F(1,14)=0, p=0.989	F(1,14)=0.37, p=0.555
Last Drinking Day	F(1,14)=0.23, p=0.639	F(1,14)=0.13, p=0.721	F(1,14)=0.02, p=0.881	F(1,14)=0, p=0.977	F(1,14)=0.97, p=0.342
Treatment * Age	F(1,14)=4.46, p=0.055	F(1,14)=2.41, p=0.144	F(1,14)=2.02, p=0.178	F(1,14)=0, p=0.964	F(1,14)=0.52, p=0.483

Supplementary Table 9 Linear Mixed Models for ALC vs CON contrast across 5 ROIs

	Bl VMPFC			L Caudate			R Caudate			L DLPFC			R DLPFC		
Predictors	β	CI	p	β	CI	p	β	CI	p	β	CI	p	β	CI	p
(Intercept)	0.22	-0.20 – 0.64	.296	-0.12	-0.52 – 0.27	.534	-0.06	-0.45 – 0.32	.747	-0.04	-0.38 – 0.29	.803	-0.11	-0.56 – 0.34	.623
Session	-0.14	-0.36 – 0.09	.227	0	-0.21 – 0.21	.999	-0.01	-0.22 – 0.19	.905	-0.07	-0.24 – 0.10	.421	-0.12	-0.33 – 0.08	.236
Treatment	-0.06	-0.33 – 0.20	.631	0.1	-0.15 – 0.34	.43	0.08	-0.17 – 0.32	.531	0.02	-0.19 – 0.22	.878	0	-0.26 – 0.27	.982
Age	0	-0.01 – 0.01	.906	0	-0.01 – 0.01	.724	0	-0.01 – 0.01	.796	0	-0.01 – 0.01	.974	0	-0.01 – 0.01	.782
Antidepressant Use	-0.13	-0.33 – 0.06	.177	-0.09	-0.28 – 0.09	.321	-0.07	-0.25 – 0.11	.451	-0.05	-0.21 – 0.10	.501	-0.05	-0.26 – 0.16	.656
Alcoholic Liver Disease	0.11	-0.09 – 0.31	.278	0.14	-0.05 – 0.33	.137	0.09	-0.09 – 0.27	.334	0.14	-0.02 – 0.30	.086	0.17	-0.05 – 0.38	.129
Session * Treatment	0.19	-0.17 – 0.54	.3	0.04	-0.29 – 0.38	.796	-0.02	-0.34 – 0.31	.912	0.12	-0.15 – 0.40	.364	0.07	-0.25 – 0.40	.651
**Random Effects**															
σ^2	0.09			0.08			0.07			0.05			0.07		
τ_{00}	0.00 ID			0.00 ID			0.00 ID			0.00 ID			0.01 ID		
ICC										0.03			0.16		
N	23 ID			23 ID			23 ID			23 ID			23 ID		
Observations	46			46			46			46			46		
Marginal R^2	0.084			0.079			0.035			0.093			0.085		

P-threshold < .029 (Bonferroni's adjustment corrected)

Note. Reference category for predictor shown in brackets. σ^2 = random effects variance; τ_{00} = random intercept variance ICC = intra-class correlation coefficient; ID = individual participants (random factor); Bl = bilateral, L = left, R = right, DLPFC = dorsolateral prefrontal cortex.

Supplementary Table 10 Sensitivity Analysis Linear Mixed Models for ALC vs CON contrast across 5 ROIs

	Bl VMPFC			L Caudate			R Caudate			L DLPFC			R DLPFC		
Predictors	β	CI	p	β	CI	p	β	CI	p	β	CI	p	β	CI	p
(Intercept)	0.25	-0.17 – 0.67	.250	-0.12	-0.52 – 0.28	.545	-0.05	-0.44 – 0.34	.802	-0.01	-0.33 – 0.31	.947	-0.08	-0.54 – 0.38	.735
Session	-0.14	-0.36 – 0.08	.227	0	-0.21 – 0.21	.999	-0.01	-0.21 – 0.19	.906	-0.07	-0.24 – 0.10	.416	-0.12	-0.32 – 0.08	.229
Treatment	-0.03	-0.30 – 0.25	.837	0.1	-0.16 – 0.36	.470	0.09	-0.16 – 0.34	.489	0.06	-0.15 – 0.27	.582	0.04	-0.24 – 0.32	.782
Age	0	-0.01 – 0.01	.690	0	-0.01 – 0.01	.736	0	-0.01 – 0.01	.914	0	-0.01 – 0.00	.609	0	-0.01 – 0.01	.973
Antidepressant Use	-0.14	-0.34 – 0.05	.148	-0.09	-0.28 – 0.09	.336	-0.07	-0.25 – 0.11	.439	-0.07	-0.22 – 0.08	.365	-0.06	-0.27 – 0.16	.605
Alcoholic Liver Disease	0.1	-0.13 – 0.34	.386	0.14	-0.08 – 0.37	.207	0.09	-0.12 – 0.31	.400	0.11	-0.07 – 0.29	.239	0.17	-0.09 – 0.43	.201
Smoker	0	-0.26 – 0.26	1.000	0	-0.26 – 0.25	.969	-0.01	-0.26 – 0.23	.913	0.05	-0.15 – 0.25	.636	-0.02	-0.31 – 0.27	.905
Last Drinking Day	0.01	-0.01 – 0.03	.410	0	-0.02 – 0.02	.956	0	-0.01 – 0.02	.736	0.01	-0.00 – 0.03	.146	0.01	-0.01 – 0.03	.439
Session * Treatment	0.19	-0.17 – 0.54	.301	0.04	-0.29 – 0.38	.800	-0.02	-0.34 – 0.31	.914	0.12	-0.14 – 0.39	.358	0.07	-0.24 – 0.39	.649
**Random Effects**															
σ^2	0.09			0.08			0.07			0.05			0.07		
τ_{00}	0.00 ID			0.00 ID			0.00 ID			0.00 ID			0.01 ID		
ICC										0.03			0.16		
N	23 ID			23 ID			23 ID			23 ID			23 ID		
Observations	46			46			46			46			46		
Marginal R^2	0.095			0.075			0.035			0.145			0.095		

Supplementary Table 11 Connections with significant seed region posterior cingulate 9 from Schaeffer-400 atlas parcellation

Seed ROI	Connection Side	Connection target	Test statistic	p-unc	p-FDR
Dorsal Attentional Posterior Cingulate 9			F(2,17)=20.75	0.000027	.010
	Right	Somatomotor A 6	T(18)=-6.13	0.000009	.003
	Right	Somatomotor A 10	T(18)=-5.06	0.000081	.013
	Left	Somatomotor A 7	T(18)=-4.95	0.000104	.013
	Left	Somatomotor A 10	T(18)=-4.76	0.000156	.015
	Left	Visual B Striate Calcarine 1	T(18)=-4.52	0.000266	.020
	Left	Somatomotor B Central 5	T(18)=-4.42	0.000332	.021
	Right	Somatomotor A 2	T(18)=-4.11	0.000657	.035
	Right	Somatomotor A 7	T(18)=-4.04	0.000765	.036
	Right	Somatomotor A 4	T(18)=-3.63	.002	.081
	Right	Temporal Parietal 2	T(18)=-3.51	.002	.093
	Right	Somatomotor A 11	T(18)=-3.47	.003	.094
	Right	Salience/Ventral Attention B Medial Posterior PFC 1	T(18)=-3.37	.003	.106
	Right	Visual B Extra-striate Superior 2	T(18)=-3.3	.004	.116
	Left	Somatomotor A 3	T(18)=-3.21	.005	.130
	Right	Visual A Extra-striate Inferior 10	T(18)=-3.16	.005	.131
	Left	Somatomotor A 13	T(18)=-3.15	.006	.131
	Right	Somatomotor A 3	T(18)=-2.98	.008	.161
	Left	Somatomotor B Central 4	T(18)=-2.94	.009	.161

	Left	Temporal Parietal 3	T(18)=-2.94	.009	.161
	Left	Visual B Striate Calcarine 2	T(18)=-2.93	.009	.161
	Right	Visual B Striate Calcarinel 1	T(18)=-2.91	.009	.161
	Left	Visual A Extrastriate 10	T(18)=-2.91	.009	.161

Supplementary Table 12 Sensitivity Analyses of connections with significant seed region posterior cingulate 9 from Schaeffer-400 atlas parcellation

Seed ROI	Connection Side	Connection target	Test statistic	p-unc	p-FDR
Dorsal Attentional Posterior Cingulate 9			F(2,15)=17.21	.000131	.049
y	Right	Somatomotor A 6	T(16)=-5.16	.000095	.036
	Left	Somatomotor A 7	T(16)=-4.52	.000347	.062
	Left	Visual B ExtraStriate Superior 6	T(16)=-4.22	.000646	.062
	Left	Somatomotor B Aud 16	T(16)=-4.13	.000787	.062
	Right	Somatomotor A 10	T(16)=-4.07	.000887	.062
	Left	Somatomotor A 10	T(16)=-4.02	.000981	.062
	Right	Somatomotor A 2	T(16)=-3.89	.001	.064
	Right	Salience/Ventral Attention B Medial Posterior PFC Medial 2	T(16)=-3.87	.001	.064
	Right	Visual A Extra-striate 11	T(16)=-3.63	.002	.095
	Left	Temporal Parietal 2	T(16)=-3.55	.003	.101
	Right	Somatomotor A 11	T(16)=-3.37	.004	.122
	Right	Visual A Extra-striate 7	T(16)=-3.37	.004	.122
	Right	Somatomotor A 7	T(16)=-3.33	.004	.122
	Right	Visual A Extra-striate 12	T(16)=-3.21	.005	.147
	Right	Dorsal Attentional Posterior Cingulate 4	T(16)=-3	.008	.213
	Right	Somatomotor B S2 8	T(16)=-2.94	.01	.214

Post Hoc Power Analyses

We conducted a post hoc power analysis for our linear mixed models (LMMs) using the methodology described by (Quach et al., 2022). This approach is particularly suitable for complex models where fixed and random effects are included. Our analysis focused on determining the power to detect the interaction between session (Session) and treatment group (Treatment) on the 5 defined regions of interest. These post hoc power analyses were thus conducted the final sample N = 23, with n= 14 in the placebo (PLA) group and n = 9 in the N-acetylcysteine (NAC) group, measured at two time points (T0, T1). An extended dataset was created to reflect this structure, ensuring that Treatment and Session were treated as factors in the model.

We fit a series of LMMs for each dependent variable ROI (L caudate, R caudate, L/R ventromedial prefrontal cortex, L dlpfc, and R dlpfc) using the formula:

$$\text{ROI} \sim \text{Session} * \text{Treatment} + \text{Age} + \text{Antipdepressant Use} + \text{Alcohol-related Liver Disease} + (1 \mid \text{subject ID})$$

The interaction term Session * Treatment was the fixed effect of primary interest.

Using the simr package, we extended the fitted models to the required sample size and conducted power simulations. The powerSim function was employed to estimate the power of detecting the interaction effect (session(T1):Treatment(NAC)). This analysis was based on Equation 9 from Quach et al.'s (2022) paper, which incorporates the observed effect size to determine the power. The equation:

$$Power = 1 - \Phi\left(z_{1-\frac{\alpha}{2}} - \frac{\sigma}{\delta}\right)$$

was effectively translated into the powerSim framework, where z represents the critical value, δ the observed effect size, and σ the standard error.

The power analysis was performed for each dependent variable ROI with 1000 simulations, for the respective ALC-ALC and CON-CON models, with the results summarized in Supplementary Table 13.

Supplementary Table 13 Post Hoc Power Analyses for ALC and CON LMM contrast models

Region	ALC contrast power (%)	CON contrast power (%)
Bl VMPFC	15.5	14.7
L Caudate	18.8	27.1
R Caudate	21.3	20.6
L DLPFC	27.4	20.5
R DLPFC	31.3	24.6

References

Abraham, A, Pedregosa, F, Eickenberg, M *et al.* (2014) Machine learning for neuroimaging with scikit-learn. *Front Neuroinform* **8**.

Avants, BB, Epstein, CL, Grossman, M, Gee, JC (2008) Symmetric diffeomorphic image registration with cross-correlation: Evaluating automated labeling of elderly and neurodegenerative brain. *Med Image Anal* **12**: 26-41.

Avants, BB, Tustison, N, Song, G (2009) Advanced normalization tools (ANTS). *Insight j* **2**: 1-35.

Ciric, R, Thomas, AW, Esteban, O, Poldrack, RA (2022) Differentiable programming for functional connectomics. *arXiv preprint arXiv:220600649*.

Ciric, R, Wolf, DH, Power, JD *et al.* (2017) Benchmarking of participant-level confound regression strategies for the control of motion artifact in studies of functional connectivity. *Neuroimage* **154**: 174-87.

Cox, RW (1996) AFNI: Software for Analysis and Visualization of Functional Magnetic Resonance Neuroimages. *Comput Biomed Res* **29**: 162-73.

Cox, RW and Hyde, JS (1997) Software tools for analysis and visualization of fMRI data. *NMR Biomed* **10**: 171-78.

Dale, AM, Fischl, B, Sereno, MI (1999) Cortical Surface-Based Analysis: I. Segmentation and Surface Reconstruction. *Neuroimage* **9**: 179-94.

Esteban, O, Ciric, R, Finc, K *et al.* (2020) Analysis of task-based functional MRI data preprocessed with fMRIPrep. *Nat Protoc* **15**: 2186-202.

Esteban, O, Markiewicz, CJ, Blair, RW *et al.* (2019) fMRIPrep: a robust preprocessing pipeline for functional MRI. *Nature Methods* **16**: 111-16.

Evans, AC, Janke, AL, Collins, DL, Baillet, S (2012) Brain templates and atlases. *Neuroimage* **62**: 911-22.

Fonov, VS, Evans, AC, McKinstry, RC, Almli, CR, Collins, DL (2009) Unbiased nonlinear average age-appropriate brain templates from birth to adulthood. *Neuroimage* **47**: S102.

Fox, PT and Lancaster, JL (2002) Opinion: Mapping context and content: the BrainMap model. *Nat Rev Neurosci* **3**: 319-21.

Greve, DN and Fischl, B (2009) Accurate and robust brain image alignment using boundary-based registration. *Neuroimage* **48**: 63-72.

Harris, CR, Millman, KJ, van der Walt, SJ *et al.* (2020) Array programming with NumPy. *Nature* **585**: 357-62.

Holla, B, Karthik, S, Biswal, J *et al.* (2018) Brain Functional Magnetic Resonance Imaging Cue-reactivity Can Predict Baclofen Response in Alcohol Use Disorders. *Clin Psychopharmacol Neurosci* **16**: 290-301.

Hunter, JD (2007) Matplotlib: A 2D Graphics Environment. *Computing in Science & Engineering* **9**: 90-95.

Jenkinson, M, Bannister, P, Brady, M, Smith, S (2002) Improved Optimization for the Robust and Accurate Linear Registration and Motion Correction of Brain Images. *Neuroimage* **17**: 825-41.

Klein, A, Ghosh, SS, Bao, FS *et al.* (2017) Mindboggling morphometry of human brains. *PLoS Comput Biol* **13**: e1005350.

Lanczos, C (1964) Evaluation of Noisy Data. *Journal of the Society for Industrial and Applied Mathematics Series B Numerical Analysis* **1**: 76-85.

Power, JD, Mitra, A, Laumann, TO, Snyder, AZ, Schlaggar, BL, Petersen, SE (2014) Methods to detect, characterize, and remove motion artifact in resting state fMRI. *Neuroimage* **84**: 320-41.

Quach, NE, Yang, K, Chen, R *et al.* (2022) Post-hoc power analysis: a conceptually valid approach for power based on observed study data. *General Psychiatry* **35**: e100764.

Reuter, M, Rosas, HD, Fischl, B (2010) Highly accurate inverse consistent registration: A robust approach. *Neuroimage* **53**: 1181-96.

Satterthwaite, TD, Elliott, MA, Gerraty, RT *et al.* (2013) An improved framework for confound regression and filtering for control of motion artifact in the preprocessing of resting-state functional connectivity data. *Neuroimage* **64**: 240-56.

Schaefer, A, Kong, R, Gordon, EM *et al.* (2018) Local-Global Parcellation of the Human Cerebral Cortex from Intrinsic Functional Connectivity MRI. *Cerebral cortex (New York, NY : 1991)* **28**: 3095-114.

Schulte, MHJ, Kaag, AM, Boendermaker, WJ, van den Brink, W, Goudriaan, AE, Wiers, RW (2019) The effect of N-acetylcysteine and working memory training on neural mechanisms of working memory and cue reactivity in regular cocaine users. *Psychiatry Research: Neuroimaging* **287**: 56-59.

Tustison, NJ, Avants, BB, Cook, PA *et al.* (2010) N4ITK: Improved N3 Bias Correction. *IEEE Trans Med Imaging* **29**: 1310-20.

Virtanen, P and Gommers, R and Oliphant, TE *et al.* (2020) SciPy 1.0: fundamental algorithms for scientific computing in Python. *Nature Methods* **17**: 261-72.

Yarkoni, T, Markiewicz, CJ, de la Vega, A *et al.* (2019) PyBIDS: Python tools for BIDS datasets. *J Open Source Softw* **4**.

Zhang, Y, Brady, M, Smith, S (2001) Segmentation of brain MR images through a hidden Markov random field model and the expectation-maximization algorithm. *IEEE Trans Med Imaging* **20**: 45-57.
